# Study problems and depressive symptoms in adolescents during the COVID-19 outbreak: poor parent-child relationship as a vulnerability

**DOI:** 10.1186/s12992-021-00693-5

**Published:** 2021-04-06

**Authors:** Jingyi Wang, Hao Wang, Haijiang Lin, Marcus Richards, Shuyue Yang, Hongbiao Liang, Xiaoxiao Chen, Chaowei Fu

**Affiliations:** 1 School of Public Health; Key Laboratory of Public Health Safety; NHC Key Laboratory of Health Technology Assessment, Fudan University, Shanghai, 200032 China; 2 Taizhou City Center for Disease Prevention and Control, Taizhou, 318000 Zhejiang Province China; 3 MRC Unit for Lifelong Health and Ageing, Institute of Cardiovascular Science, University College London, London, UK

**Keywords:** COVID-19, Adolescents, Depression, Study problem, Parent-child relationship

## Abstract

**Background:**

Little is known about the prevalence of and risk factors for adolescent mental health problems during the COVID-19 outbreak. We aimed to investigate the prevalence of depressive symptoms, their association with study-relevant problems, and the moderating effect of parent-child relationship among Chinese adolescents during the school closures.

**Methods:**

We performed a cross-sectional analysis with data collected in middle and high schools in Taizhou, China. Students completed an online survey between April 16 and May 14, 2020. Depressive symptoms were assessed using the Children’s Depression Inventory. Three types of study problems were recorded, including having difficulty in studying at home, dislike of remote learning, and excessive screen entertainment time. Parental relationships were categorized into good or normal relationship and poor relationship. Linear regression and logistic regression analyses were conducted to investigate the associations between study-relevant problems and depressive symptoms.

**Results:**

Using data from 6435 adolescents, we found that the prevalence of depressive symptoms was 17.7%. All the study problem measures were associated with more severe depressive symptoms. There was a moderating effect of the parental relationship on the associations between study problems and depressive symptoms. The association between number of study problems and depressive symptoms was stronger in adolescents with a poor parent-child relationship (regression coefficient 4.34 [95% CI 2.97, 5.72]) than those with a good or normal relationship (2.55 [2.35, 2.75]), p for interaction 0.002, on multivariable adjustment.

**Conclusions:**

Study problems due to school closures were particularly problematic for adolescents who had poor parent-child relationships. Public health initiatives could help students to adjust study habits and improve parent-child relationships, thereby protecting against the development of depression.

**Supplementary Information:**

The online version contains supplementary material available at 10.1186/s12992-021-00693-5.

## Introduction

The coronavirus disease 2019 (COVID-19) pandemic has caused an unparalleled disruption of education worldwide [1]. As of June 28, 2020, nationwide school closures had been implemented in 116 countries, and localized closures in many other countries according to estimations of the United Nations Educational, Scientific and Cultural Organization [2]. Consequently, over 1 billion young people (62% of total enrolled learners) are currently out of school education [2].

Although school closures are often used as a way of reducing transmission during infectious disease outbreaks, the adverse impact of prolonged school closures on mental health of the adolescents involved should be noted [3]. Amidst the pandemic period, many students struggle to adaptation to major changes in study routines, e.g. remote learning at home and excessive screen entertainment time [4]. These changes may interfere with sense of structure, security and self-efficacy, and predispose young people to negative emotional impacts of a stressful situation [5]. Consistent support from parents or carers can serve as a buffer against these effects [6]. While most children eventually return to their typical functioning if there is a supportive and responsive caregiver, others may not recover from harmful effects of the pandemic [7]. For example, during this time of social distancing, adolescents in an abusive home are at greater risk of developing mental health problems such as depression [8].

There are a strikingly small number of studies reporting the prevalence of mental health problems and their risk or protective factors in children and adolescents during the pandemic period [9]. A preprint study among the first community quarantined in the United States found that 40.1% of parents observed signs of manageable distress in their children, and 5.5% reported signs of significant distress [10]. The ongoing Co-SPACE survey in the UK reported an increase in childhood behavioral and restless or attentional difficulties over 1 month as lockdown progressed, although there was no change in emotional difficulties [11]. A study in Hubei province during COVID-19 outbreak reported elevated depressive (22.6%) and anxiety symptoms (18.9%) among Chinese primary school children [12]. Similarly, Zhou and colleagues [13] reported 43.7% mild to severe depressive symptoms in junior and senior high school students in China. However, as the studies only tested associations with sociodemographic characteristics, it remains unclear how school closures affect student mental health. A better understanding of pandemic-related risk factors can help optimize interventions in the mental health of children in countries affected by COVID-19. In terms of the parent-child relationship during the pandemic, limited studies reported that the raised parenting stress negatively affected parents’ relationship with their children and increased the use of harsh parenting [14], while parenting self-efficacy might be a protective factor for their children’s emotional well-being [15]. However, the two studies only involved young children and did not investigate the effect of parent-child relationship on the associations between mental health and study problems which may be especially important to adolescents.

We conducted a cross-sectional analysis to investigate the prevalence of depressive symptoms and their association with study-relevant problems among Chinese adolescents in Taizhou during the school closures. We also aimed to examine the role of poor parent-child relationship as a critical factor moderating the link between adolescent study problems and depressive symptoms.

## Methods

### Participants

In this population-based cross-sectional study, data were collected in middle and high schools in Taizhou, a city in the Zhejiang Province in China, between April 16 and May 14, 2020. Cluster sampling was adopted, and 12 middle schools and 12 high schools were randomly selected, which covered key, ordinary and private schools. Two classes were randomly selected from each grade in each school. All students in the selected classes were invited to participate in the online survey through the Wenjuanxing platform (https://www.wjx.cn). A total of 7242 students provided written informed consent and completed questionnaires. After excluding 655 invalid questionnaires and 152 with missing data for age, 6435 participants were included in the analyses. For full detail see the additional figure [see Additional file 1]. This study was approved by the Research Ethics Committee of the School of Public Health, Fudan University (IRB#2020040817).

### Assessment of depression

Depressive symptoms were measured by the 27-item Children’s Depression Inventory (CDI) [16]. Modelled on the Beck Depression Inventory, this self-rating scale was adapted to measure the cognitive, affective, somatic and behavioral signs of depression in people 7–17 years of age. Each item in the CDI consists of three statements scored from 0 to 2, and the child is asked to choose the best statement that describes their feelings and thoughts during the last 2 weeks. Total scores range from 0 to 54, with higher scores indicating more severe depressive symptoms. A cutoff score of ≥19 for categorizing depression was recommended for the general population [16]. The Chinese version of CDI was reported to have good validity and reliability [17], and showed excellent internal consistency in our sample (Cronbach’s α = 0.90).

### Assessment of study problems and parent-child relationship

The questions about study problems asked participants whether they currently had difficulty in studying at home; whether they had difficulty in studying before school closures; whether they like remote learning; and the average hours of screen entertainment time per day. Screen entertainment time was classified as < 8 h/day or ≥ 8 h/day. The number of study problems was summed over the three types of problems (difficulty in studying at home, dislike of remote learning, screen entertainment ≥8 h/day). Relationships with mothers and fathers were recorded (Is your relationship with your mother good, normal or poor? Is your relationship with your father good, normal or poor?), and the responses of the two questions were categorized into good or normal relationship vs. poor relationship with either the mother or father. Study and parent-child relationship problems were summed and categorized into four groups (no problem of either type, study problems only, parent-child relationship problems only, study and parent-child relationship problems).

### Assessment of sociodemographic and pandemic characteristics

Information regarding individual sociodemographic and pandemic characteristics included: sex, age, economic status, type of school, father’s and mother’s educational attainment, whether relatives and friends died or had serious illness, collective and home quarantine experience, and whether the individual was nervous or anxious about the pandemic.

### Statistical analysis

Participant characteristics were reported as mean (SD) for continuous data and as frequency and percentage within each category for categorical variables. Linear regression was used to calculate regression coefficients and 95% confidence intervals (CIs) for 1) the association between each study problem and depression score stratified by relationship with mother and father; and 2) the association between number of study problems and depression score stratified by relationship with parents. Interactions between study problems and parent-child relationships were tested. Model 1 adjusted for sex and age (Having difficulty studying at home was additionally adjusted for having difficulty studying in school); model 2 added sociodemographic factors (economic status, school type, mother’s or father’s education); and model 3 additionally adjusted for pandemic characteristics (relatives or friends died or with serious illness, quarantine experience, and feelings about the pandemic). Sensitivity analyses were conducted using logistic regression for associations between study and parent-child relationship problems and depression as a binary outcome. Odds ratios (ORs) and 95% CIs were reported. All analyses were conducted in Stata version 15.1 (StataCorp LP, College Station, TX).

## Results

The mean (SD) age of the participants was 15.6 (1.7) years, and 50.2% were female. Adolescents with a poor parent-child relationship had lower economic status and father’s education, and experienced more collective and home quarantine compared to those with good or normal parent-child relationship. The mean (SD) of CDI total score was 11.2 (7.4) for adolescents with good or normal parent-child relationship and 19.9 (9.9) for those with a poor relationship. The frequency of depression (CDI ≥ 19) was 17.7% in the whole sample, and was higher in those with poor parent-child relationship than in the good or normal group (52.4% vs 16.3%). The participants with a poor parent-child relationship had more study problems, including difficulty in studying, dislike of remote learning, and screen entertainment≥8 h/day (Table 1).

**Table 1:** Characteristics of the study population

	Total (*n* = 6435)	Good or normal parent-child relationship (*n* = 6183)	Poor parent-child relationship (*n* = 252)
**Sociodemographic and pandemic variables**			
Female, n (%)	3231 (50.2)	3114 (50.4)	117 (46.4)
Age, years	15.6 (1.7)	15.6 (1.7)	15.4 (1.6)
Economic status, n (%)			
High	651 (10.1)	634 (10.3)	17 (6.7)
Middle	5348 (83.1)	5168 (83.6)	180 (71.4)
Low	436 (6.8)	381 (6.2)	55 (21.8)
Key school, n (%)	2438 (37.9)	2344 (37.9)	94 (37.3)
Father’s education, n (%)			
Primary school or lower	1195 (18.6)	1137 (18.4)	59 (23.4)
Middle/high school	4537 (70.5)	4364 (70.6)	173 (68.7)
College or higher	702 (10.9)	682 (11.0)	20 (7.9)
Mother’s education, n (%)			
Primary school or lower	1617 (25.1)	1552 (25.1)	65 (25.8)
Middle/high school	4147 (64.4)	3985 (64.5)	162 (64.3)
College or higher	671 (10.4)	646 (10.4)	25 (9.9)
Relatives/friends died or with serious illness, n (%)	374 (5.8)	357 (5.8)	17 (6.7)
Collective/home quarantine, n (%)	1378 (21.4)	1306 (21.1)	72 (28.6)
Nervous/anxious about the pandemic, n (%)	3138 (48.8)	3020 (48.8)	118 (46.8)
**Depressive symptoms measures**			
Total Score for the Children’s Depression Inventory (CDI) (range 0–54)	11.5 (7.7)	11.2 (7.4)	19.9 (9.9)
CDI ≥ 19, n (%)	1140 (17.7)	1008 (16.3)	132 (52.4)
**Study problems**			
Having difficulty in studying at home, n (%)	3490 (54.2)	3300 (53.4)	190 (75.4)
Having difficulty in studying in school, n (%)	2857 (44.4)	2707 (43.8)	150 (59.5)
Dislike remote learning, n (%)	2967(46.1)	2803 (45.3)	164 (65.1)
Screen entertainment ≥8 h/day, n (%)	733 (11.4)	692 (11.2)	41 (16.3)

Data are mean (SD) or n (%)

Among participants with a poor relationship with the mother, having difficulty in studying at home was associated with a higher depression score compared to those without this study difficulty (regression coefficient 8.32 [95% CI 3.66, 12.99] in model 1) (Table 2). The association was weaker in those with a good or normal relationship with the mother (regression coefficient 4.35 [95% CI 3.94, 4.76]). Additional adjustment for sociodemographic and pandemic factors made no major difference. In the fully adjusted model 3, the regression coefficients were 7.44 [95% CI 2.45, 12.43] in participants with a poor mother-child relationship and 4.13 [95% CI 3.72, 4.54] in those with a good or normal relationship, respectively. In addition, among participants with a poor relationship with the mother, dislike of remote learning (regression coefficient 5.72 [95% CI 1.49, 9.96]) and excessive screen entertainment time (≥8 h/day) (regression coefficient 3.19 [95% CI -2.86, 9.24]) were associated with a higher depression score (model 3), although the confidence limits were wider for screen entertainment time. The regression coefficients for students with a good or normal relationship were smaller (regression coefficient 2.14 [95% CI 1.78, 2.50]; 2.34 [95% CI 1.77, 2.91]). Associations between having difficulty in studying at home, dislike of remote learning and depressive symptoms differed by mother-child relationship, when tested as an interaction (*p* < 0.05). Similarly, associations between the three forms of study problems and depressive symptoms were stronger in the group with a poor relationship with the father than the group with a good or normal relationship (Table 3).

**Table 2:** Associations between study problems and depression score stratified by relationship with mother

	Regression coefficient and 95% CI	
	Good or normal relationship with mother (***n*** = 6315)	Poor relationship with mother (***n*** = 120)
**Having difficulty in studying at home**		
M1: sex, age and having difficulty in studying in school	4.35 (3.94 to 4.76)	8.32 (3.66 to 12.99)
M2: M1 + economic status, school type and mother’s education	4.21 (3.80 to 4.62)	8.26 (3.45 to 13.07)
M3: M2 + relatives or friends died or with serious illness, quarantine experience and feelings about the pandemic	4.13 (3.72 to 4.54)	7.44 (2.45 to 12.43)
**Dislike remote learning**		
M1: sex and age	2.24 (1.87 to 2.61)	4.99 (0.95 to 9.03)
M2: M1 + economic status, school type and mother’s education	2.16 (1.79 to 2.52)	4.87 (0.70 to 9.03)
M3: M2 + relatives or friends died or with serious illness, quarantine experience and feelings about the pandemic	2.14 (1.78 to 2.50)	5.72 (1.49 to 9.96)
**Screen entertainment ≥ 8 h/day**		
M1: sex and age	2.59 (2.01 to 3.17)	3.42 (−2.32 to 9.16)
M2: M1 + economic status, school type and mother’s education	2.41 (1.83 to 2.98)	3.62 (−2.38 to 9.62)
M3: M2 + relatives or friends died or with serious illness, quarantine experience and feelings about the pandemic	2.34 (1.77 to 2.91)	3.19 (−2.86 to 9.24)

Using multivariable linear regression analyses with depression score as dependent variable and study problems as independent variables, stratified by relationship with mother. Positive regression coefficients reflect more severe depressive symptoms

**Table 3:** Associations between study problems and depression score stratified by relationship with father

	Regression coefficient and 95% CI	
	Good or normal relationship with father (***n*** = 6261)	Poor relationship with father (***n*** = 174)
**Having difficulty in studying at home**		
M1: sex, age and having difficulty in studying in school	4.27 (3.86 to 4.68)	7.97 (4.42 to 11.51)
M2: M1 + economic status, school type and father’s education	4.14 (3.73 to 4.55)	8.22 (4.63 to 11.80)
M3: M2 + relatives or friends died or with serious illness, quarantine experience and feelings about the pandemic	4.07 (3.66 to 4.48)	8.25 (4.62 to 11.87)
**Dislike remote learning**		
M1: sex and age	2.23 (1.86 to 2.60)	3.58 (0.38 to 6.79)
M2: M1 + economic status, school type and father’s education	2.16 (1.80 to 2.53)	3.56 (0.28 to 6.84)
M3: M2 + relatives or friends died or with serious illness, quarantine experience and feelings about the pandemic	2.15 (1.79 to 2.51)	4.17 (0.83 to 7.52)
**Screen entertainment ≥ 8 h/day**		
M1: sex and age	2.52 (1.94 to 3.10)	3.30 (−0.88 to 7.47)
M2: M1 + economic status, school type and father’s education	2.34 (1.77 to 2.92)	3.13 (−1.10 to 7.36)
M3: M2 + relatives or friends died or with serious illness, quarantine experience and feelings about the pandemic	2.27 (1.70 to 2.85)	3.12 (−1.14 to 7.39)

Using multivariable linear regression analyses with depression score as dependent variable and study problems as independent variables, stratified by relationship with father. Positive regression coefficients reflect more severe depressive symptoms

**Fig. 1:**
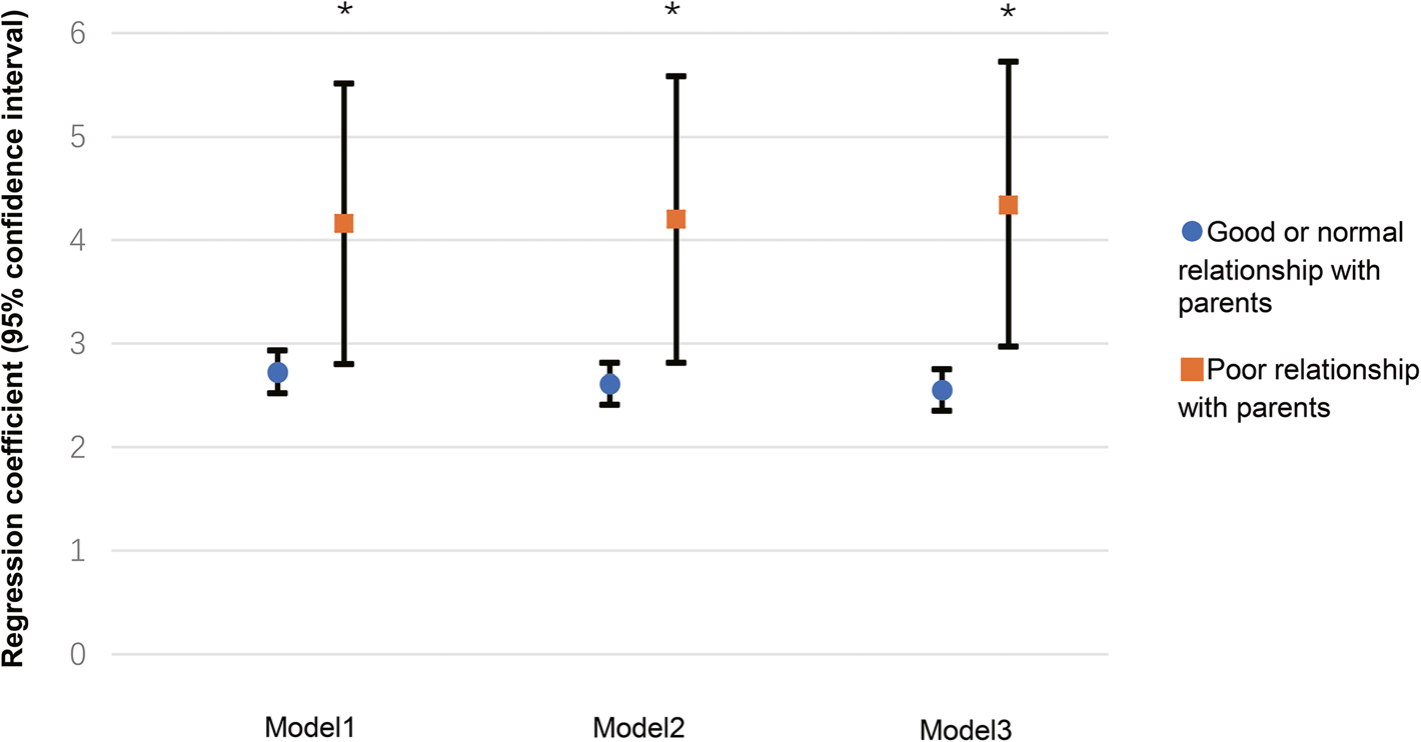
The effect sizes in the association between number of study problems and depression score stratified by relationship with parents in linear regression. Model 1 was adjusted for sex and age. Model 2 was additionally adjusted for economic status, school type, mother’s education and father’s education. Model 3 was adjusted for model 2 plus relatives or friends died or with serious illness, quarantine experience and feelings about the pandemic. *p < 0.01 for interaction number of study problems - relationship with parents

Participants with more study problems were likely to have worse depressive symptoms. Associations were stronger in the group with a poor parent-child relationship (regression coefficient 4.34 [95% CI 2.97, 5.72]) than the group with a good or normal relationship (regression coefficient 2.55 [95% CI 2.35, 2.75]) in fully adjusted model 3. The number of study problems × parent-child relationship interaction was strong (regression coefficient 1.63 [95% CI 0.59, 2.66], *p* = 0.002) (Fig. 1).

Sensitivity analyses using CDI score of 19 or higher as a threshold for elevated depressive symptoms yielded similar findings. While students with study problems only (OR 3.21 [95% CI 2.62, 3.92]) or with parent-child relationship problems only (OR 2.11 [95% CI 0.78, 5.67]) were more likely to have depression than those with no problem of either type, depression was much more likely to occur in participants who had both study and parent-child relationship problems than those without any problems (OR 16.25 [95% CI 11.68 to 22.62] in model 3) (Table 4).

**Table 4:** Associations between study problems and parent-child relationship problems and depression (binary outcome)

Study/parent-child relationship problems	Odds ratio and 95% CI (***n*** = 6435)		
	M1: sex and age	M2: M1 + economic status, school type, mother’s education and father’s education	M3: M2 + relatives/friends died or with serious illness, quarantine experience and feelings about the pandemic
No problem of either type	Reference		
Study problems only	3.35 (2.75 to 4.08)	3.26 (2.67 to 3.98)	3.21 (2.62 to 3.92)
Parent-child relationship problems only	2.51 (0.95 to 6.64)	2.18 (0.82 to 5.84)	2.11 (0.78 to 5.67)
Both study and parent-child relationship problems	18.37 (13.28 to 25.40)	16.69 (12.00 to 23.19)	16.25 (11.68 to 22.62)

Using multivariable logistic regression analyses with dichotomized depression as dependent variable and study/parent-child relationship problems as independent variable. Odds ratios indicate the likelihood of having depression for people with a type of study/parent-child relationship problems

## Discussion

In this cross-sectional epidemiological study, we found that the prevalence of depressive symptoms in middle and high school students in Taizhou was 17.72%. This is lower than the figures found in the other two studies among primary school students (22.6%) [12] and high school students (43.7%) [13] in China after the outbreak of COVID-19. However, the two studies were either conducted in Hubei province or included a large number of participants in Hubei province where the infection rate of COVID-19 was highest in China. The studies among general population in China reported that young people and students had higher levels of depression and experienced a greater psychological impact of the pandemic [18, 19]. It is also notable that the prevalence of depression in our sample was much higher in adolescents with poor parent-child relationship (52.38%) than those with good or normal relationship (16.30%). This result was consistent with previous studies which found that poor parent-child relationship and growth in family conflict increased the risk of adolescent depressive symptoms [20, 21].

The results of our study provided substantial support for findings of associations between having difficulty in studying at home and dislike of remote learning and depressive symptoms. They also showed some evidence for the association between excessive screen entertainment time and depressive symptoms, although the results should be interpreted with some caution due to the wide confidence limits in participants with poor parent-child relationship. These associations were independent of sociodemographic and pandemic risk factors. One apparent question concerns how these study problems adversely affect adolescent mental health. Students who had difficulty in studying at home may worry about their academic performance. A large number of studies have shown that adolescents’ poor academic performance was closely associated with a high prevalence of depression [22–24]. Remote learning has been reported to have several disadvantages, e.g. lack of self-discipline and self-motivation, harder to understand content when not face-to-face with teachers, and sense of isolation [25], which could harm mental health. Excessive screen use displaces time participating in healthier activities [26], imposes too much upward social comparison through social media [27, 28], and immerses adolescents in negative information consistent with their biased cognitions [28]. Alternatively, participants may have been influenced by depression already before they suffered from study problems. As a matter of fact, depressive symptoms affect adolescents’ capability to pursue education and are often accompanied by addictive behaviors [29]. Our study also found that as the number of study problems increased, adolescent depressive symptoms also increased. Thus, the accumulation of difficulty in studying at home, dislike of remote learning and excessive screen entertainment time is related to more severe depressive symptoms in adolescence.

In terms of the moderating effect of parent-child relationship on the associations between study problems and depressive symptoms, our results indicated that study problems due to school closures were particularly problematic for adolescents who had a poor relationship with the mother or father. Although previous studies have reported the separate effects of study problems and parent-child relationship on adolescent depressive symptoms [23, 28, 30], our study adds an important component by suggesting that the combination of the two substantially increased risk for triggering or precipitating depressive symptoms. Study problems and parent-child relationship have been associated with personal qualities such as self-esteem, which is closely related to the development of depression, suggesting that impaired self-esteem may underlie both types of difficulties [20, 28]. Additionally, during the experience of study problems, adolescents may rely on their relationship with mother or father for security and reassurance, which then offsets their emotional disturbances [31]. However, poor parental interaction styles, e.g. being highly critical, frequent child comparison, discouraging expression of opinions and invalidating ideas, may become potent stressors for students who have already experienced study difficulties [32]. Moreover, our results indicated that, in addition to mother-child relationship, the role of fathers was important as well, since perception of the father-child relationship exerted a moderating effect on the relationship between study problems and depressive symptoms. Although we cannot rule out the possibility that parent-child relationship might mediate the associations between study problems and depressive symptoms, analyses using the data from our sample did not provide enough support for this effect (data not shown). More longitudinal research is needed to untangle the direction of effect in the association between study problems, parent-child relationship and depression.

Due to the COVID-19 epidemic, schools at all levels were shut down in China and adolescents’ lifestyles have greatly changed thereafter. Although education authorities have developed online courses and other learning materials, the various restrictions imposed on daily life such as isolation at home and the potential adverse influence on academic development have posed a major threat to adolescents’ mental health [33]. This study built on previous literature by examining three types of study problems in relation to depressive symptoms during school closures. Our findings also expanded previous work by suggesting the parent-child relationship as an important moderator that can help explain the transmission of study difficulties into the development of depression.

Several limitations of our study deserve comment. First, the cross-sectional design makes it impossible to test the direction of causality. Covariates at one time point do not take into account cumulative or past exposure, although difficulty in study at school before the pandemic was included in the models. Second, our study is unable to investigate the long-lasting impact of school closures on psychological wellbeing. Longitudinal studies are needed to better understand the longer-term consequences of COVID-19 on mental health for children and adolescents, and to determine the mechanisms that explain the occurrence of psychological problems, including changes in psychological (e.g., self-efficacy and loneliness) [5, 34], physiological (e.g., sleep and nutrition) [35, 36] and structural (e.g., daily routines) risk factors [37]. Third, the level of depressive symptoms using self-report measure might not be consistent with the evaluation of mental health professionals. Forth, relationships with mothers and fathers were just measured by two items and the nature of parent-child relationship might not be sufficiently captured by single items.

## Conclusions

In conclusion, our findings showed that when students had study problems during the pandemic outbreak, those with poor parent-child relationship were particularly vulnerable to experiencing increases in depressive symptoms. Our findings suggested that caregivers, researchers and clinicians need to be aware of the negative impact of study problems and the potential role of a positive parent-child relationship in countering such stress during school closures. Interventions that help students adjust study habits and improve parent-child relationship may reduce stress and relieve emotional disturbances. If school closures are essential, then our results suggest that officials and policy makers should take measures to protect adolescents at high risk of mental health problems which may be caused by lifestyle changes, domestic conflicts and tension with parents [38].

## Supplementary Information

**Additional file 1: Figure S1.** Flow chart showing sample size included in the study.

## Data Availability

The data and code that support the findings of this study are available from the corresponding author (CF) upon reasonable request.
