# Supplementary figures and images for: Study problems and depressive symptoms in adolescents during the COVID-19 outbreak: poor parent-child relationship as a vulnerability
Source: Global Health. 2021 Apr 6;17:40. doi: 10.1186/s12992-021-00693-5 (PMC8022312; doi:10.1186/s12992-021-00693-5)

**Figure S1. Flow chart showing sample size included in the study**

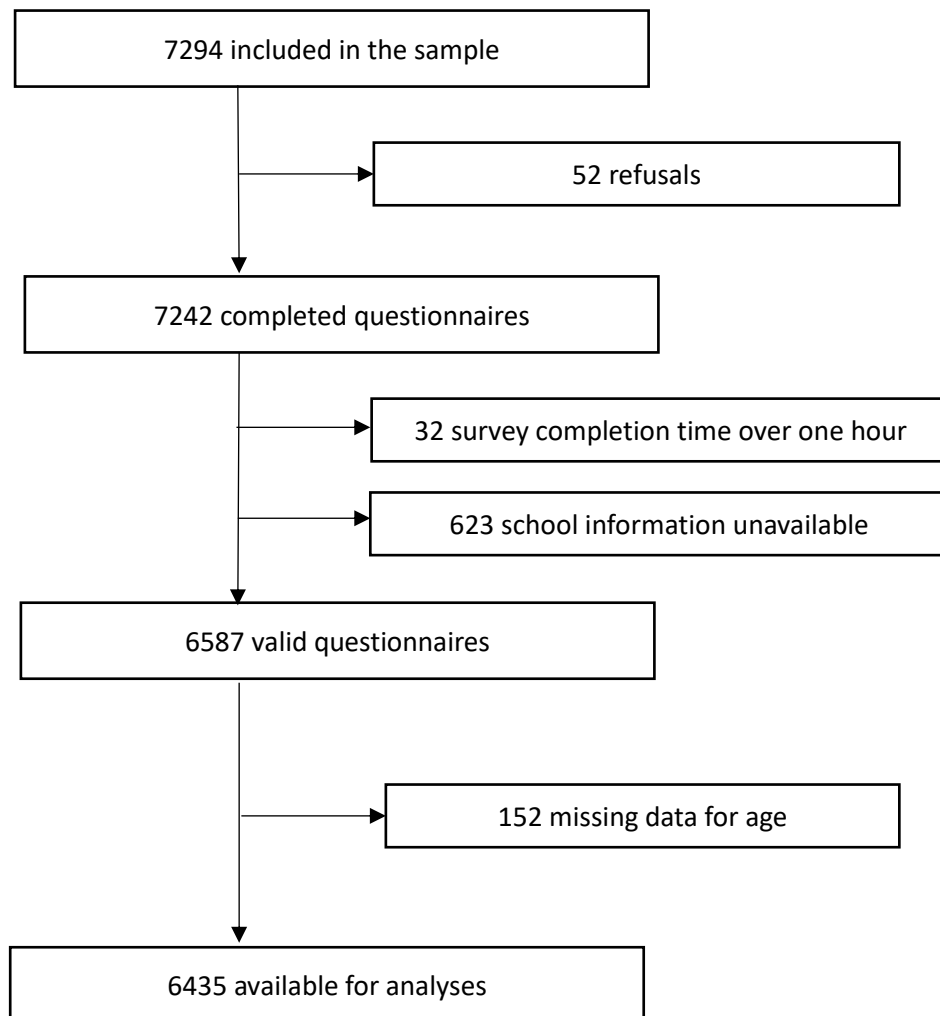

Supplement: Supplementary file 1 — Additional file 1: Figure S1. Flow chart showing sample size included in the study. [file 12992_2021_693_MOESM1_ESM.pdf]
